# Chondroitin Sulfate/Polycaprolactone/Gelatin Electrospun Nanofibers with Antithrombogenicity and Enhanced Endothelial Cell Affinity as a Potential Scaffold for Blood Vessel Tissue Engineering

**DOI:** 10.1186/s11671-021-03518-x

**Published:** 2021-04-17

**Authors:** Xiangqian Kong, Yuxiang He, Hua Zhou, Peixian Gao, Lei Xu, Zonglin Han, Le Yang, Mo Wang

**Affiliations:** Vascular Surgury, Shandong Provincial Hospital Affiliated to Shandong First Medical University, Jinan, 250021 China

**Keywords:** Blood vessel tissue engineering, Gelatin/polycaprolactone nanofiber, Chondroitin sulfate, Endothelial cells

## Abstract

Electrospun polymer nanofibers have gained much attention in blood vessel tissue engineering. However, conventional nanofiber materials with the deficiencies of slow endothelialization and thrombosis are not effective in promoting blood vessel tissue repair and regeneration. Herein, biomimetic gelatin (Gt)/polycaprolactone (PCL) composite nanofibers incorporating a different amount of chondroitin sulfate (CS) were developed via electrospinning technology to investigate their effects on antithrombogenicity and endothelial cell affinity. Varying CS concentrations in PG nanofibers affects fiber morphology and diameter. The CS/Gt/PCL nanofibers have suitable porosity (~ 80%) and PBS solution absorption (up to 650%). The introduction of CS in Gt/PCL nanofibers greatly enhances their anticoagulant properties, prolongs their coagulation time, and facilitates cell responses. Particularly, 10%CS/Gt/PCL nanofibers display favorable cell attachment, elongation, and proliferation. Thus, the Gt/PCL nanofibers containing a certain amount of CS could be excellent candidates as a promising tissue-engineering scaffold in blood vessel repair and regeneration.

## Introduction

The development of nanofibrous materials have elicited much attention as biomimetic scaffolds in tissue repair and regeneration owing to their unique (bio)physicochemical features, including their extracellular matrix (ECM)-like ultrafine fiber structure, excellent mechanical properties, large specific surface area, and high porosity with interconnectivity [1, 2]. It has been demonstrated that nanofibrous structures play a key role in mediating cellular responses, such as cell adhesion, morphology (e.g., spreading, alignment, elongation, etc.), arrangement, migration, proliferation, phenotype, and differentiation via contact guidance [3–5]. While numerous nano-fabrication strategies (e.g., nanoskiving, template synthesis, phase separation, etc.) have provided an available technology to fabricate nanofibrous materials, electrospinning is one of the most effective methods to fabricate nanofibers from different materials (metals, ceramic, polymer, composite materials, etc.) on an industrial scale [6–8].

In addition to (bio)physical signals, the selection of suitable biomaterials can significantly affect cell activities as well as tissue repair and regeneration [9]. Some key features, such as biocompatibility, bioresorbability, mechanical properties, and bio-function, should be considered [9, 10]. Compared to natural/natural and synthetic/synthetic composites, composite nanofiber materials consisting of natural and synthetic polymers have received more attention owing to the combination of the excellent biological features of natural polymers and the mechanical strength of synthetic polymers [9]. Among them, the gelatin (Gt)/polycaprolactone (PCL) combination is one of the most representatively investigated natural-synthetic hybrid systems and is widely used in blood vascular tissue engineering [11–14]. However, Gt/PCL composite nanofibers still have some limitations, such as slow endothelialization, and thrombosis. Recently, chondroitin sulfate (CS) is a sulfated polysaccharide containing glycosaminoglycan and galactosamine, which has been demonstrated to possess high adhesion to endothelial cells (ECs), weak interaction to proteins and platelets, as well as electrostatic repulsion of negatively charged blood components [15–17]. In addition, CS could inhibit cellular apoptosis and facilitate the healing of vascular wounds [18, 19]. Therefore, Gt/PCL (PG) electrospun nanofibers incorporating CS would be excellent candidates as a bio-instructive tissue-engineering scaffold for blood vascular repair and regeneration.

In the present work, biomimetic PG composite nanofibers containing different CS ratios were developed via one-step electrospinning. The morphology, chemical feature porosity, and degradation and of CS/PG composite nanofibers were detected by different characterization techniques. The anticoagulant of PG/CS composite nanofibers was evaluated. Further, these composite nanofibers with different CS ratios were seeded with human aortic endothelial cells (HAECs) to investigate their effects on cellular responses.

## Materials and Methods

### Materials

CS (bovine trachea, type A, purity: 95%) was supplied by Shanghai Macklin Biochemical Technology Co., Ltd. (China). PCL was obtained from Aladdin Biochemical Polytron Technologies Inc. (China). Gt (bovine skin, type B) were obtained from Sigma-Aldrich Biochemical Technology Co., Ltd., (China). The activated partial thromboplastin time (APTT) kit was purchased from Leigen Biotechnology Co., Ltd (China). Acetic acid (purity: 99.5%) was purchased from Sinopharm Chemical Reagent Co., Ltd. (China). The human aortic endothelial cells (HAECs) were obtained from the affiliated hospital of Qingdao University (China). Culture medium Dulbecco's Modified Eagle Medium/Nutrient Mixture F-12 (DMEM/F-12), fetal bovine serum (FBS), 0.25% trypsin–EDTA were purchased from Biological Industries (Israel). All reagents were purchased from Sigma Aldrich (China) unless otherwise noted. The water used in all experiments was deionized.

### Electrospinning of Nanofibers

PCL (10% w/v) was dissolved in acetic acid under mechanic stirring at room temperature for 4 h. Gt (10% w/v) was dissolved in 90% acetic acid with constant stirring for 2 h. CS in different concentrations was added into the Gt solution and stirred gently at room temperature for 1 h to obtain the homogenous solutions containing 5, 10, and 15 wt.% CS relative to the total polymer concentration. Then, PG solution was prepared by mixing two above solutions in a weight ratio of 50/50 (w/w) under stirring for 2 h, named as 5%CS@PG, 10%CS@PG, and 15%CS@PG.

The prepared homogenous solutions were subjected to the electrospinning process, equipped with a 1 mL syringe with 21 G needle and a collector covered by aluminum foil. In this study, the distance between the collector plate and the needle tip was fixed at around 18 cm, the voltage was set at 23 kV, and the polymeric solutions were pumped out at a rate of 1 mL/h. All solutions were electrospun in an electrospinning instrument (Technology, Tk602TH, China) at room temperature and carefully controlled humidity (< 40%). Prior to any further experiments, the samples were put in a vacuum drying oven for 72 h at least to remove any remaining solvent.

### Scanning Electron Microscopy (SEM) and Transmission Electron Microscope (TEM)

The morphology of CS@PG nanofibrous scaffolds was investigated by an SEM (VEGAS, TESCAN, Czech) at an acceleration voltage of 20 kV at room temperature. The diameter of nanofibers (*n* = 100) was further measured from SEM images using image analysis software (Image J).

The TEM observations and energy-dispersive X-ray spectroscopy (EDS) analyses were carried out using a JEOL JEM-2100 plus (Japan).

### Fourier Transform Infrared Spectroscopy (FTIR)

FTIR was carried out by a Nicolet iN10 FTIR spectrometer (Thermo Fisher Scientific, Waltham, MA, USA) to evaluate the characteristic functional groups of CS, PG nanofiber, and CS@PG nanofibers. The spectra of samples were recorded with the transmission mode over a wavelength range of 4000–500 cm^−1^ with a resolution of 2 cm^−1^.

### Porosity and the Absorption of Phosphate-Buffered Saline (PBS) Solution

The porosity of nanofibers was carried out using the liquid displacement method. Firstly, the dry weight of nanofibers was weighed as W_1_. Then, four groups of nanofibers were immersed in ethanol for 2 h at 25 °C and weighted as W_2_. The ethanol on the sample surface was removed by filter paper, and the weights of samples were then noted as W_3_. The porosity of the nanofibers was calculated by the following formula:

$${\text{Porosity}}\left( \% \right) = \left( {W_{3} - W_{1} } \right)/\left( {W_{3} - W_{2} } \right) \times 100$$

For the PBS solution absorption test, the obtained nanofibers were weighed in a dry state and recorded as Wd. Then, the nanofibers were soaked in PBS for 24 h at 25 °C and the weight at wet state was recorded as Ww after removing the excess liquid on the sample surface. The swelling ratio can be measured by the following equation:

$${\text{PBS}}\;{\text{absorption}}\left( \% \right) = \left( {W_{{\text{W}}} - W_{{\text{d}}} } \right)/W_{{\text{d}}} \times 100$$

All the values in the above experiments are expressed as the mean ± SD (*n* = 3).

### In Vitro Degradability Behavior

To determine the obtained nanofibers’ resistance to lysozyme, the degradability of the samples was measured at a scheduled time (1, 4, 7, 10, and 14 days). The initial weight of the nanofibers was recorded as W_i_. Then, the samples were immersed in PBS solution (pH 7.4) containing lysozyme (500 μg/mL) and incubated in vitro in 37 °C. At the predetermined degradation intervals, each group of samples was removed and washed by deionized water, and freeze-dried to obtain the final weight (*W*_f_). The lysozyme solution was changed three times a week. The mass remaining (%) of the nanofibrous scaffolds was estimated as defined in the following formula:

$${\text{Mass}}\;{\text{remaining}}\left( \% \right) = \left( {1 - \frac{{w_{i} - w_{f} }}{{w_{i} }}} \right) \times 100\%$$

To evaluate the degradation behavior of the sample after 7 days, the morphology of nanofibers was observed by SEM.

### Blood Compatibility Analysis

#### Coagulation Times

Activated partial thromboplastin time (APTT) was used to assess the activity of intrinsic and common pathways of coagulation through testing the clotting times of platelet-poor plasma (PPP) after incubation with electrospun nanofibers on coverslips. For this, anticoagulated blood (approximately 10 mL) was collected and centrifuged at 3000 rpm for 20 min to obtain platelet-poor plasma (PPP). Each sample was incubated with 200 μL of PPP for 10 min at 37 °C and analyzed using APTT kit following the manufacture’s instruction (*n* = 3).

#### Hemolysis Testing

The hemolysis rate was evaluated by measuring the concentration of hemoglobin released into the solution phase from erythrocytes in diluted whole blood exposed to the electrospun nanofibers. The samples on coverslips were individually placed into 24-well plates and immersed in 2 mL PBS at 37 °C for 30 min. The negative control groups contained only 2 mL of PBS, while positive control groups were comprised of 2 mL deionized water for the aim of inducing maximal lysis of erythrocytes (*n* = 3 for each testing group). Then, 40 μL of aforementioned anticoagulated fresh whole blood was added into each well and incubated for 60 min at 37 °C, after which the suspensions were removed into centrifuge tubes and centrifuged at 100× *g* for 5 min. The supernatants were subjected to measure the absorbance at 570 nm using a microplate reader (SynergyH1/H1M, BioTek, China).

#### Thrombogenicity

The thrombogenic potential was evaluated in vitro after incubation of electrospun samples with the platelet-rich plasma (PRP). PRP was prepared by centrifugation (1500 rpm, 20 min) and the upper one-half of plasma was discarded. Then, the nanofibers on coverslips were incubated in 100 μL of PRP at 37 °C for 2 h and gently rinsed with PBS three times for subsequent experiments. To evaluate the platelet activity after being incubated with PRP, electrospun nanofibers were kept in the DMEM supplemented with 10% FBS for 2 h and 24 h, and then a CCK-8 assay kit was utilized to measure thrombocyte viability on the nanofibers. CCK-8 (20 μL) was added to 200 μL of serum-free DMEM medium in 24-well plates and incubated for 1 h. Finally, suspensions were measured by a microplate reader at a wavelength of 450 nm.

### Cell Culture and Cell Viability

Human aortic endothelial cells (HAECs) were cultured in DMEM supplemented with FBS (10%, v/v), and streptomycin/penicillin (1%, v/v) in an atmosphere of 37 °C and 5% CO_2_. The culture medium was replaced three times a week. The cells were generally isolated by 0.05% trypsin/EDTA for 3 min, centrifuged at 1000 rpm, and suspended in the fresh medium to carry out cell passage or cell seeding.

Prior to cell seeding, all the nanofibrous materials in a 24-well plate were sterilized under UV irradiation for 1 h and immersed in 75% ethanol (v/v, %) for 1 h. Then, the nanofibers were rinsed four times with PBS solution and soaked in DMEM for 12 h in CO_2_ incubator. The cells were seeded at a density of 1 × 10^4^ cells per well on nanofibers and the medium was replaced every day. After co-cultured 24 h, cells were washed by PBS solution three times and then stained with a Calcein-AM/PI Double stain Kit (YEASEN Biochemical Technology Co., Ltd., Shanghai, China). PI was used to stain dead cells and Calcein-AM stained for living cells.

#### Cell Morphology on the Nanofibers

To observe the cell adhesion on the nanofibers, the morphology of the cells was observed after co-cultured 24 h by Fluorescence Microscope (Nikon A1 MP, Japan). Firstly, the cells were fixed with 4% paraformaldehyde at room temperature for 30 min. Then, 0.5% Triton X-100 solution was used for 5 min to permeabilize the cell membrane. Finally, 4′6-diamidino-2-phenylindole (DAPI) was used to stained cell nuclei and rhodamine-phalloidin was used to stain the F-actin. Quantitative analysis (cell density, single-cell area, cell elongation) was further performed with Image J.

#### Cell Proliferation

Cell proliferation activity on the composite nanofibers was carried out with a CCK-8 Assay Kit. The HAECs were seeded on nanofibers at a density of 8 × 10^3^ cells/well. The culture medium was changed every two days. After 1, 4, 7 days of co-culturing, the cells were washed by PBS solution to remove non-adherent cells. Then, 20 μL CCK-8 and the complete medium at a volume ratio of 1: 10 was added into the 24-well plate for 1 h at the incubator. The absorbance was tested by Elisa Reader at 450 nm.

Cell staining with DAPI and rhodamine-phalloidin was performed with Fluorescence Microscope to observed directly the changes of cell counts after co-cultured with different nanofibers for two days and six days.

#### Statistical Analysis

All samples in the experiments were processed in triplicate. All data are represented as means ± standard deviations (SD). Statistical analysis of samples was determined by one-way analysis variance (ANOVA) to compare differences with significance assigned at *p* < 0.05.

## Result and Discussion

### Preparation and Characterization of CS@PG Nanofibers

**Fig. 1:**
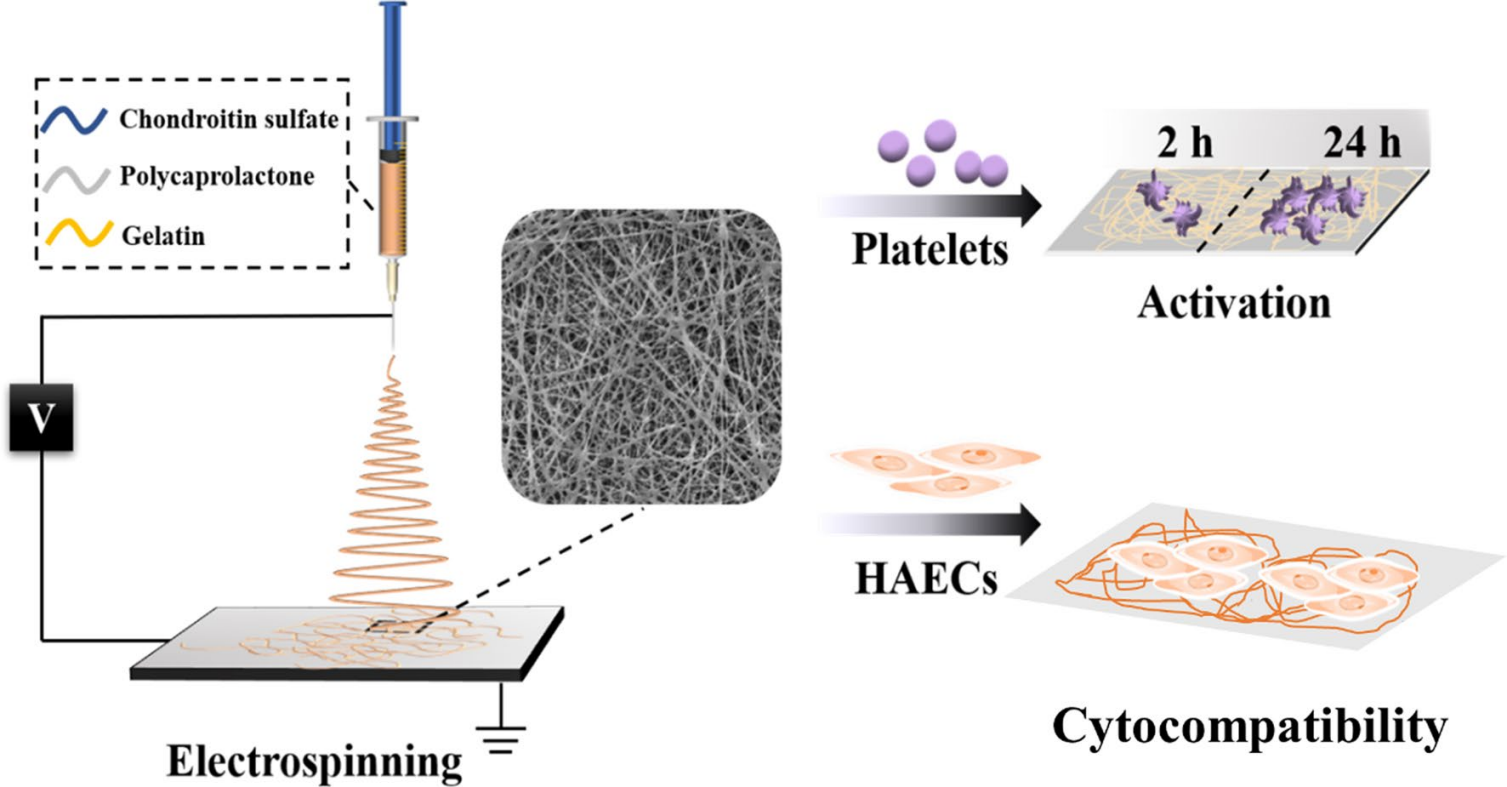
Schematic illustration of the fabrication of nanofibers and in vitro evaluation

Figure 1 displayed the schematic diagram of the electrospinning process for CS@PG nanofibrous scaffolds and in vitro evaluation of anticoagulation and cytocompatibility. To develop engineered vascular tissue scaffolds for promoting the proliferation of endothelial cells, different ratios of CS (5, 10, and 15 wt%) were incorporated into PG (10%, v/v) solutions to fabricated composite nanofibers via electrospinning process.

**Fig. 2:**
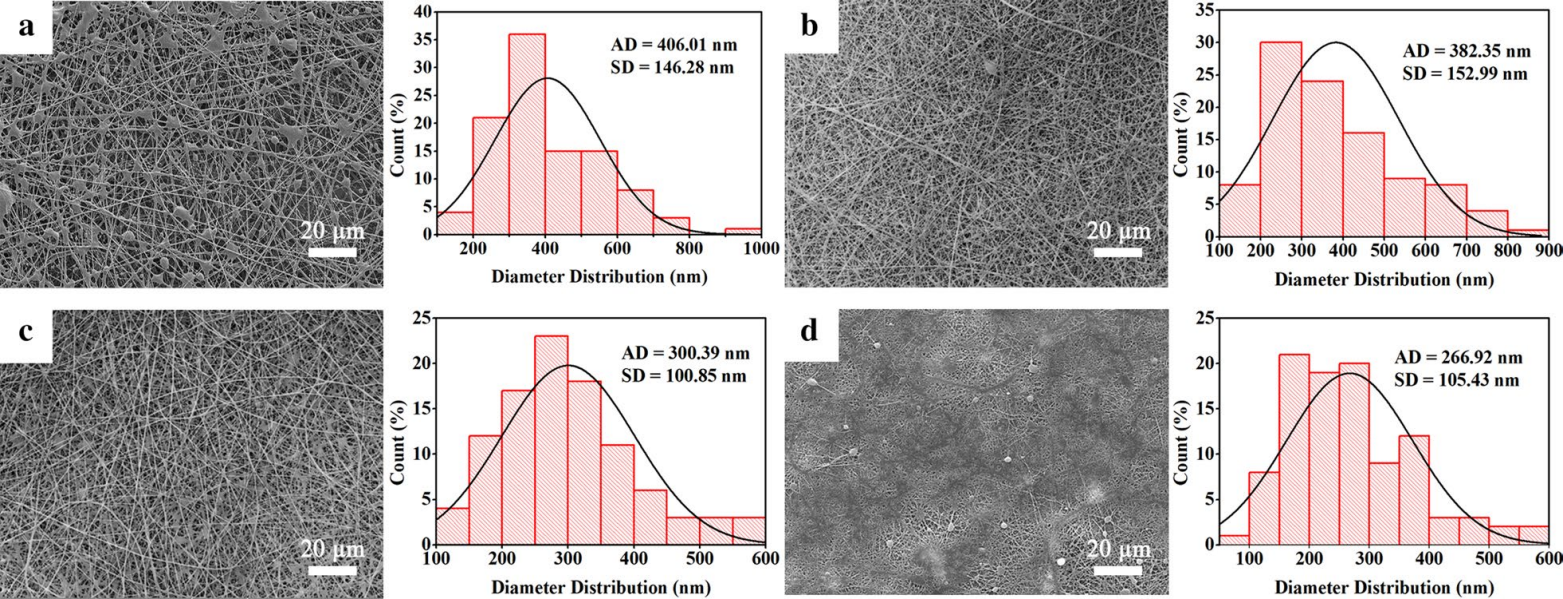
SEM images and diameter distribution of CS@PG electrospun scaffolds at different ratios of CS. **a** PG, **b** 5%CS@PG, **c** 10%CS@PG, **d** 15%CS@PG

SEM was performed to observe the structure of CS@PG electrospun nanofibers. As shown in Fig. 2, the SEM images of PG, 5%CS@PG, 10%CS@PG, 15%CS@PG nanofibers exhibited the dense fibrous architecture, containing continuous, smooth, and nano-scaled fibers by electrospinning technique. The PG nanofibers (Fig. 2a) displayed many irregular beads on nanofibers through the process of electrospinning, having an average diameter of 406.01 ± 146.28 nm. It might be explained that 10% w/v of electrospun solution exhibited low viscosity, resulting in the formation of inhomogeneous nanofibers containing beads [20]. With increasing the amount of CS in the polymeric solution from 5 to 10% wt., homogenous nanofibers without any beads could be achieved due to the increased solution viscosity (Fig. 2b, c). Correspondingly, 5%CS@PG, and 10%CS@PG showed a smaller average diameter of 382.35 ± 152.99 nm and 300.29 ± 100.85 nm, respectively. This outcome is primarily because the increase of CS content correspondingly enhanced the conductivity of electrospinning solution [20]. However, when the polymer concentration was up to 15%, the viscosity of the polymer solution is too high to be stretched in the electric field. At the same flow rate of 1 mL/h as the previous groups, we observed that under the applied voltage of 23 kV, the needle tip obstruction phenomenon appeared and the Taylor cone could not be observed. Hence, the flow rate of the electrospun solution and the applied voltage were adjusted to 0.9 mL/h and 20 kV, respectively. The 15%CS@PG nanofibers were successfully produced with an average diameter of 266.92 ± 105.43 nm.

**Fig. 3:**
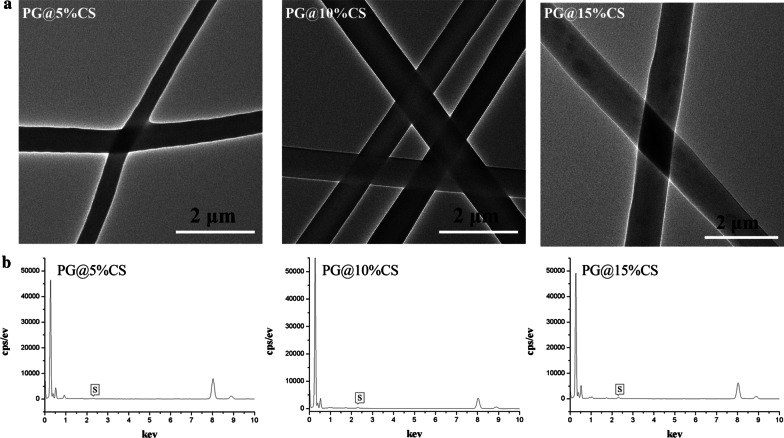
TEM images (**a**) and EDX elemental analysis of CS@PG electrospun scaffolds at different ratios of CS, i.e., PG, 5%CS@PG, 10%CS@PG, and 15%CS@PG

As depicted in Fig. 3a, it was found that CS was uniformly distributed in the 5%CS@PG and 10%CS@PG nanofibers. However, CS aggregation was detected in the 15%CS@PG fibers. As indicated in Fig. 3b, EDX elemental analysis was performed to detect the sulfur content of prepared fibers. As expected, the sulfur content increased with an increased concentration of CS in obtained fibers.

### FTIR Spectra Analysis

FTIR spectra analysis was carried out to determine the characteristic absorption peaks of the chemical group of prepared nanofibrous scaffolds. The FTIR spectrum of PG and CS were considered as the control group to compare with CS@PG nanofiber groups. In PG spectrum, strong absorption bands that appeared at 3300 cm^−1^ and 2935 cm^−1^ can be attributed to the –NH_2_ and –OH stretching vibration, and CH_2_ asymmetrical stretching, respectively. Several characteristic absorption peaks were appeared at 1652 cm^−1^ (amide I) for C=O stretching vibration, 1542 cm^−1^ (amide II) for N–H bending vibration, 1441 cm^−1^ for CH_2_ stretching, and 1267 cm^−1^ (amide III) for C–N stretching vibration [21, 22]. The CS spectrum performed characteristic absorption band at 1245 cm^−1^, representing the S=O stretching vibration in negatively charged SO_4_^2−^ [23, 24].

**Fig. 4:**
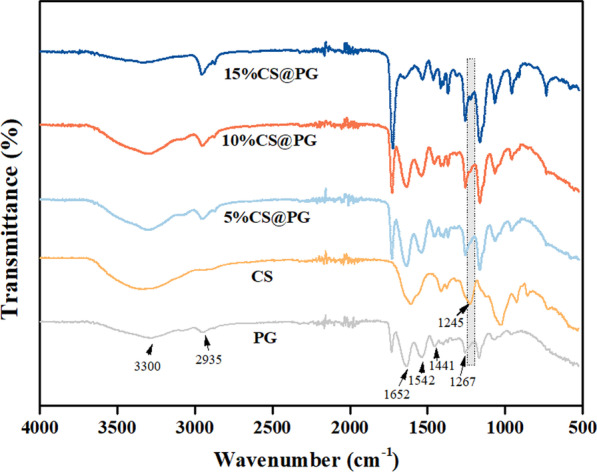
FT-IR spectrum of the CS@PG electrospun scaffolds

As shown in Fig. 4, the FTIR spectra of 5%CS@PG, 10%CS@PG, and 15%CS@PG nanofibers showed characteristic peaks of PCL, Gt, and CS together. Compared with the spectra of PG group, CS@PG groups showed the characteristic band of CS at 1245 cm^−1^, and the intensity of CS band increased by the presence of CS in nanofibers. This finding confirmed the presence of varying contents of CS in the CS@PG electrospun nanofibers.

### Porosity, Swelling Ratio, and Degradability of Nanofibers

**Fig. 5:**
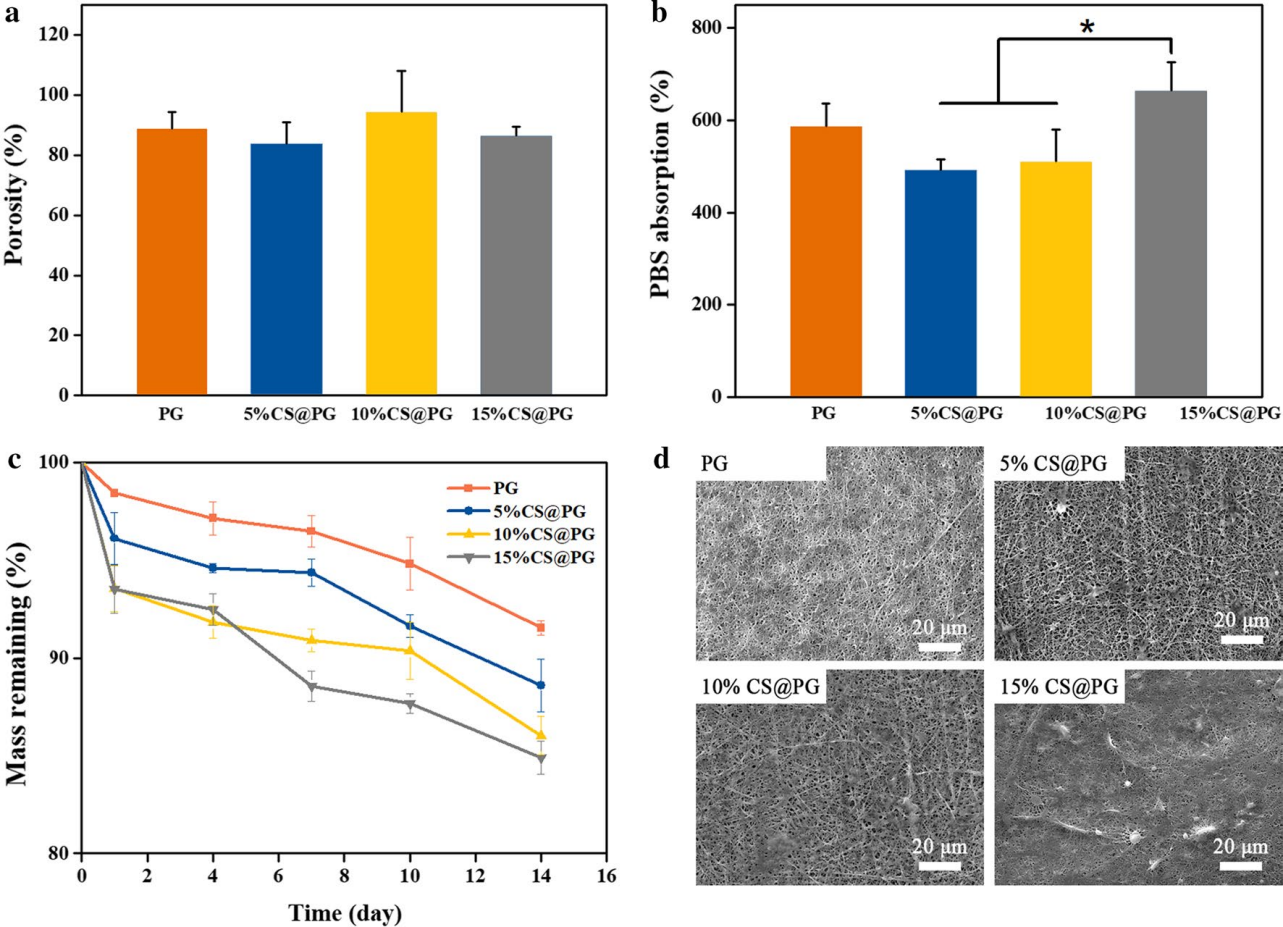
**a** The porosity of prepared nanofibers. **b** PBS absorption of prepared nanofibers. **c** Mass remaining of CS@PG nanofibers after degradation in PBS solution for up to 14 days. **d** SEM micrographs of nanofibrous films in PBS solution at 37 °C for 7 days

The high porosity of the fiber indicates that the material has excellent interconnectivity, which is conducive to the diffusion of oxygen and nutrients into the pores and promote cell infiltration [25, 26]. Figure 5a displayed the porosity of nanofibrous films. The porosity of obtained nanofibers containing different CS ratios was approximately up to 80%. The high porous architecture obtained by electrospun nanofibers can mimic the natural ECM, thus providing a favorable 3D microenvironment for cells [27]. It has been reported that scaffolds in tissue engineering with a porosity up to 80% was beneficial to the transportation of nutrients and metabolites.

The PBS absorption ratio of the CS@PG nanofibrous scaffolds was compared to evaluate the effect of CS on the PBS uptake of the films. As indicated in Fig. 5b, the PBS absorption ratio of the PG scaffold was 586.34 ± 49.44%, and the 5%CS@PG and 10%CS@PG nanofibers were 492.86 ± 21.99% and 510.04 ± 69.55%, respectively. The PBS absorption ratio of 15%CS@PG groups (665.07 ± 59.81%) was significantly higher than that of 5% CS@PG and 10%CS@PG. This result indicated that the increased content of CS in the composite nanofibers could improve the PBS uptake property of the films, which could be due to the high hydrophilicity in CS molecules containing hydrophilic groups (carboxyl and hydroxyl) [28].

The data of in vitro degradability experiment was depicted in Fig. 5c, and it was found that with the increase of CS ratio, the degradation rate of CS@PG nanofibrous scaffolds was also increased. This might be attributed the presence of SO_4_^2−^ groups [29]. As demonstrated in Fig. 5c, the degradation rate of most scaffolds began to increase after 7 days, and the degradation results at day 14 were 8.45%, 11.39%, 13.97%, and 15.10% for PG, 5% CS@PG, 10%CS@PG, and 15%CS@PG nanofibrous scaffolds, respectively. The SEM images of nanofibrous scaffolds (Fig. 5d) showed slight swelling after immersing in PBS solution for 7 days. The SEM images indicate that the developed nanofibers were stable enough to avoid serious degradation and expansion for up to 7 days, thus affecting cell response in vivo or in vitro.

### Hemocompatibility Evaluation

**Fig. 6:**
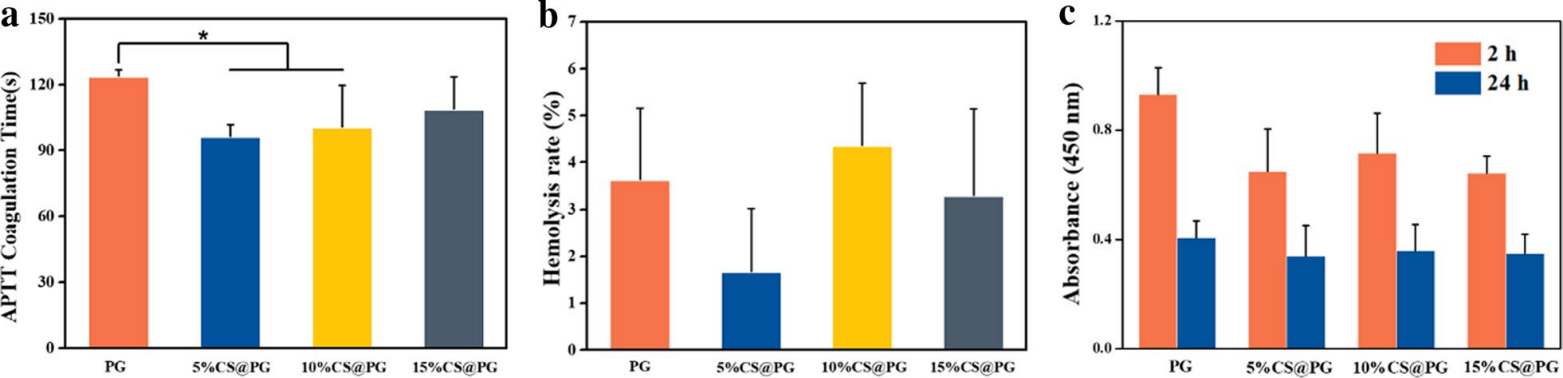
**a** APTT of CS@PG nanofibers. **b** Hemolysis rate of the samples. **c** Platelet metabolic activity on the nanofibers. *n* = 3, *p < 0.05

APTT is a simple and reliable method for detecting the blood or plasma via the intrinsic coagulation mechanism. APTT was tested to measure the influence of the electrospun nanofibers on the possible delay of blood coagulation. Clotting time detected by APTT displayed a statistically significant decrease of coagulation times between control PG and 5%CS@PG and 10%CS@PG nanofibers. In contrast, prolonged APTT was found after incubation of 15%CS@PG nanofibers with PPP, indicating its improved anticoagulant feature. As presented in Fig. 6a, the impact of blood coagulation was dependent on the diameter of nanofiber samples. The lowest APTT coagulation time was found after interaction with 5%CS@PG nanofibrous and the highest APTT with electrospun 10%CS@PG, similarly to the degree of hemolysis. The coagulation time was prolonged with the increase of CS ratio.

According to the ASTM F756-00 (2000), the hemolysis rate of the biomaterials is required to be < 5% [30]. The degree of hemolysis in this study was performed using a colorimetric assay of released hemoglobin from erythrocytes and can be caculated by the equation: Hemolysis (%) = (OD_sam_ − OD_neg_)/(OD_pos_ − OD_neg_) × 100%. As indicated in Fig. 6b, all samples were non-hemolytic and did not result in any marked hemolysis (hemolysis rate is less than 5%).

The data of platelet viability experiment are shown in Fig. 6c. After 2 h, the metabolic activity of attached thrombocytes was the highest with maximal values reached after interaction of PRP with nanofibers, particularly PG nanofibers. Interestingly, after 24 h, the platelet activity reduced in all samples. This result could be due to the rapid thrombocyte activation by the structure of nanofibers, resulting in fast consumption and releasing of growth factors compared to stable and long-term platelet activation upon contact with the smooth surface [31]. The normal life span of thrombocytes is approximately 8–10 days, and it will be shortened as platelets start to activate.

### Live/Dead Cell Staining

The influence of CS on the cytocompatibility of the nanofibers was explored by cell viability, adhesive morphology, and proliferation of HAECs evaluation onto nanofibers for 6 days. The attached cells were assessed using the Calcein-AM/PI double stain kit, DAPI staining, and CCK-8 assay kit.

**Fig. 7:**
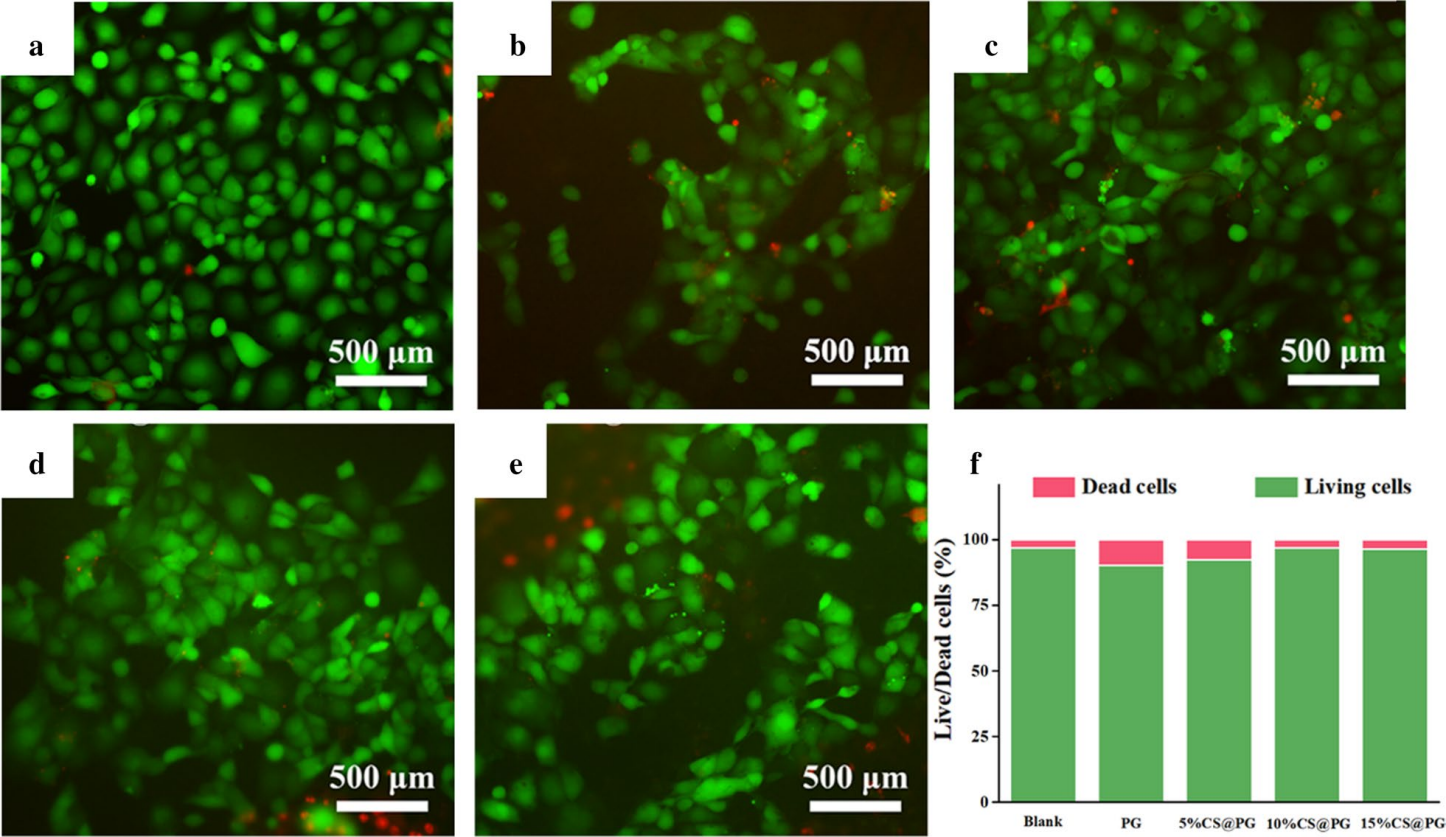
Cell viability of HAECs on the CS@PG scaffolds. **a** Tissue culture plate (TCP), **b** PG, **c** 5%CS@PG, **d** 10%CS@PG, **e**15%CS@PG, **f** The percentage of Live/Dead cell numbers of different nanofibrous scaffolds, *n* = 3

Figure 7a–e showed the fluorescence images of live and dead HAECs attached to the different nanofibers. Calcein-AM (green) and PI (red) were used to staining the live cells and dead cells, respectively. It could be observed that live cells were adhered on the CS@PG composite nanofibers, indicated that the nanofibers containing different ratios of CS had no toxicity to HAECs. Further quantitative analysis of the live/dead cell number on the nanofibers was examined by Image J (Fig. 7f). Compared with the proportion of live and dead cells on PG nanofibers, the other three groups of PG containing different CS ratios, especially 10%CS@PG and 15%CS@PG groups exhibited a larger percentage of living cells, and the percentage was increased with the increase of CS concentration. This outcome showed that the presence of CS in the nanofibers was advantageous to maintaining cell viability [32].

### Cell Adhesion Behavior on Nanofibers

**Fig. 8:**
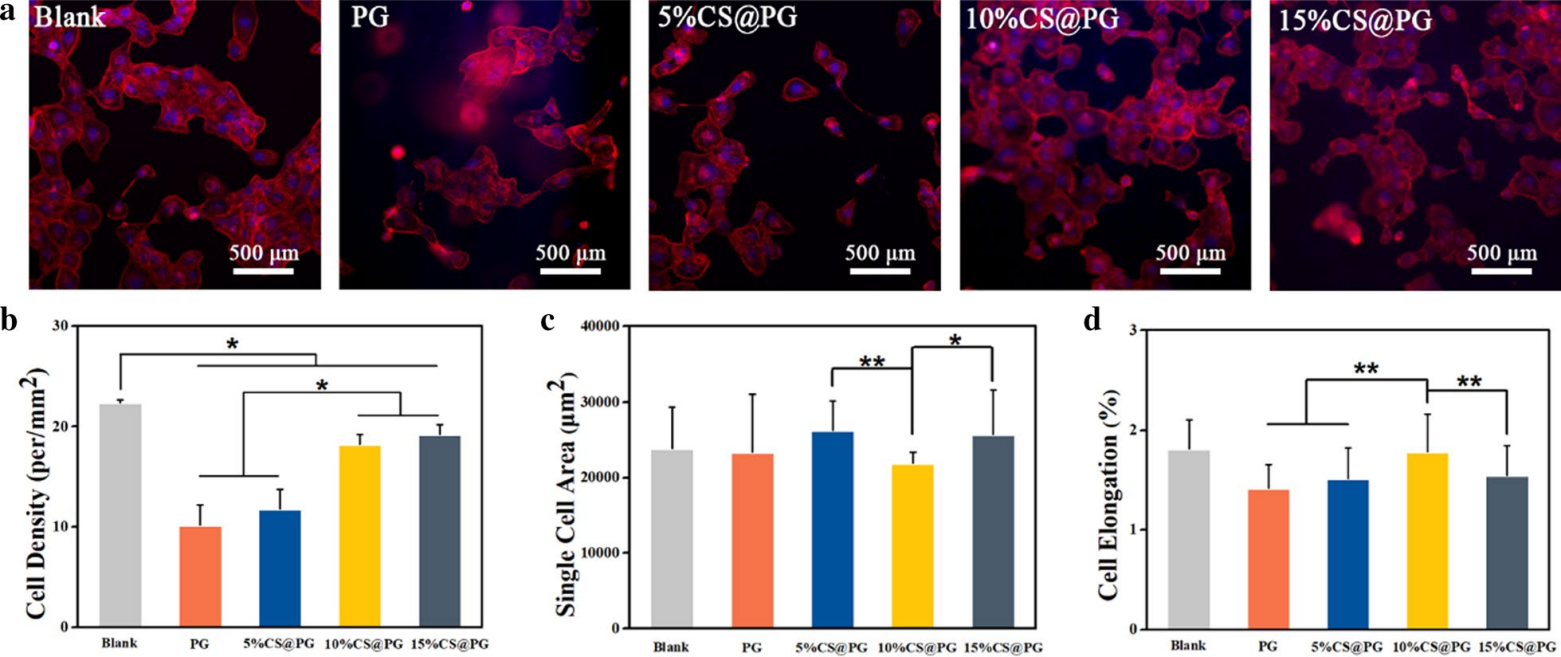
**a** Cell adhesive morphology of HAECs on the CS@PG scaffolds (DAPI: cell nuclei; Rhodamine-Phalloidin: cytoskeleton). **b** Cell density. **c** Single cell area, **d** Cell elongation in different groups of nanofibers

Nanofibrous scaffolds with good biocompatibility are considered to be the primary requirement for the generation of blood vessels. PCL with good biodegradability and natural Gt with excellent biocompatibility were widely used in tissue engineering with different structures, including hydrogels, electrospun nanofibers, and three-dimensional scaffolds [21, 33, 34]. The morphology of HAECs on the composite nanofibers and cell-materials interaction at 24 h was observed by fluorescence microscope. The cell density 10%CS@PG and 15%CS@PG was statistically significant than PG and 5%CS@PG, indicated that the addition of CS at a higher level promotes cell adhesion. As shown in Fig. 8c, the single-cell area of 5%CS@PG and 15%CS@PG was larger than the other groups of nanofibers. However, the cell elongation of 10%CS@PG was significantly higher than PG, 5%CS@PG, and 15%CS@PG (Fig. 8d). This outcome might be explained that the addition of CS at 10% concentration can facilitate the spreading of HAECs. It was found that the HAECs could adhere, spread, and grow well on the composite nanofibers, especially 10%CS@PG and 15%CS@PG. Thus, these two nanofiber materials can be considered as a safe and promising candidate vascular material in clinical application.

### Cell Proliferation

**Fig. 9:**
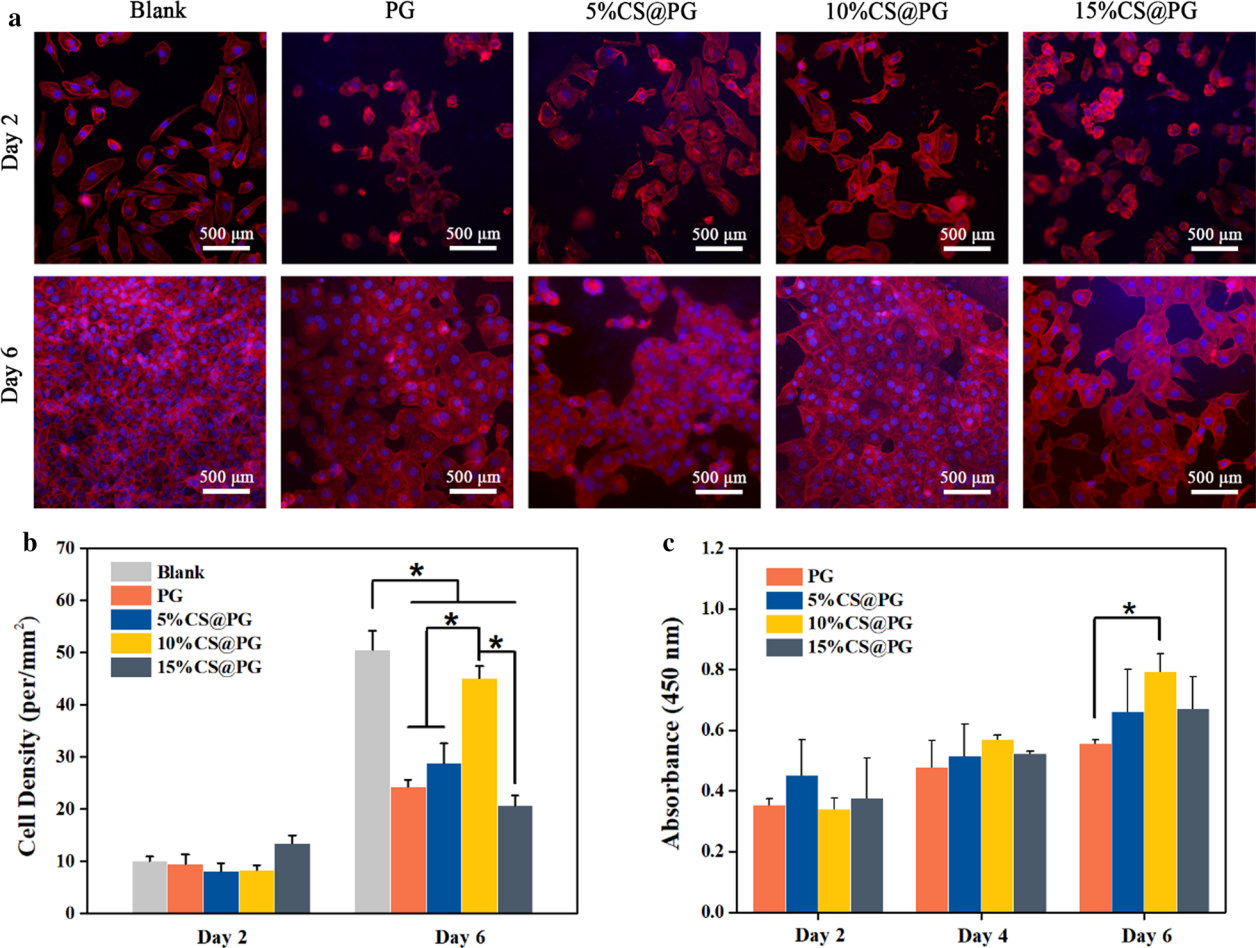
**a** HAECs proliferated on the nanofibers detected by cell staining for 2, 6 days, **b** Cell density on the nanofibrous scaffolds at 2, 6 days, *n* = 3, *p < 0.05, **c** CCK-8 assay after HAECs cultured on the PG, and CS@PG nanofibers at 2, 4, 6 days

Cell growth and viability on CS@PG nanofibers were investigated in vitro by fluorescence microscope and CCK-8 test (Fig. 9a–c). The electrospun PG scaffold was selected as the positive control. The results of the CCK-8 assay for different nanofibrous films are displayed in Fig. 9c. In 2, 4, 6 days of cells culturing, the cell number increased with the culture time for all groups. On day 6, the OD value of 10%CS@PG nanofibers was significantly higher than that of the PG scaffold. The cell proliferation comparison of 5%CS@PG, 10%CS@PG, and 15%CS@PG indicated that the cell proliferation rate was increased as the CS presence in composite nanofibers in a certain range. However, the cell proliferation capability was also influenced by the surface morphology of nanofibrous materials. Since the 15%CS@PG nanofiber exhibited strong viscosity due to the higher CS ratio in electrospinning polymeric solution, cell proliferation might be affected.

## Conclusions

In summary, biomimetic CS@PG composite nanofibers were successfully fabricated using electrospinning technology. Changed fiber morphology and diameter were obtained by varying CS concentrations in PG nanofibers. The CS@PG nanofibers possessed appropriate porosity (~ 80%) and PBS solution absorption (up to 650%). The incorporation of CS in PG nanofibers significantly improved their anticoagulant properties, prolonged their coagulation time, and enhanced cellular responses. Particularly, 10%CS@PG nanofibers exhibited favorable cell adhesion, elongation, and proliferation. Therefore, the PG composite nanofibers incorporated with a certain amount of CS inhibited antithrombogenicity and enhanced endothelial cell responses, which could be developed as a promising tissue-engineering scaffold in blood vessel repair and regeneration.

## Data Availability

All data generated or analyzed during this study are included within this article.
